# Pregnancy after advanced ovarian cancer with spontaneous uterine rupture in second trimester: A case report and review of the literature

**DOI:** 10.1002/ijgo.15837

**Published:** 2024-08-01

**Authors:** Stefan Lukac, Robin Wenzel, Fabienne Schochter, Ulrike Friebe‐Hoffmann, Beate Hüner, Wolfgang Janni

**Affiliations:** ^1^ Department of Obstetrics and Gynecology University Hospital Ulm Ulm Germany

**Keywords:** advanced ovarian cancer, fertility‐preserving surgery, second trimester, spontaneous uterine rupture

## Abstract

Fertility‐preserving surgery (FPS) in advanced ovarian cancer (AOC) is extremely rare and consequently, information about the pregnancies of these patients is anecdotal. Therefore, management of the pregnancy after AOC is challenging, especially if an unexpected situation arises. A 31‐year‐old nulliparous woman was admitted to our tertiary hospital in the 18th week of twin pregnancy with sudden severe abdominal pain. Her medical history included a low‐grade AOC stage IIIc diagnosed 2 years before pregnancy and treated by debulking FPS and systemic therapy with carboplatin/paclitaxel and bevacizumab. Clinical examination described normal vital signs and peritoneal irritation without any vaginal discharge. Sonography revealed free fluid in the pouch of Douglas and intact twin pregnancy. Laboratory work showed elevated leukocytes with neutrophilia. To evaluate appendicitis magnetic resonance imaging of the abdomen was indicated. This revealed a uterine rupture with the now extra‐cavitary position of the twins. Simultaneously, the patient's symptoms deteriorated, and emergency surgery was necessary where hemoperitoneum with avital fetuses were present. Despite excessive blood loss the uterus could be repaired and preserved. Previous resection of the uterine serosa during her debulking FPS, administration of bevacizumab affecting smooth muscles, and overstretching the uterus in the twin pregnancy were considered as possible risk factors for the presenting uterine rupture. Pregnancy after AOC is possible but should be monitored closely, especially due to the hidden long‐term consequences of its therapy. In the differential diagnosis of sudden abdominal pain during pregnancy uterine rupture should be considered even in patients with an unscared uterus.

## Synopsis

We present a case report of a spontaneous uterine rupture in the second trimester in a patient with a history of advanced ovarian cancer and fertility‐preserving surgery.

## 1 INTRODUCTION

Ovarian cancer has the worst overall survival of all gynecologic malignancies at approximately 50%.^1^ Hence, radical debulking surgery with complete resection and adjuvant chemotherapy including carboplatin, paclitaxel, and bevacizumab are applied in order to provide the best possible treatment.^2^ Advanced ovarian cancer (AOC) in women of reproductive age is rare and even rarer are the fertility‐sparing procedures in patients with AOC.^3^, ^4^ Moreover, the fertility in patients with AOC after adjuvant chemotherapy can be compromised and consequently, the knowledge about pregnancy outcomes in patients after surgical and systemic treatment in AOC is scarce.

Therefore, we report such a case from our department complicated with another rarity: a spontaneous uterine rupture in the second trimester. Additionally, we performed a narrative review of the literature for spontaneous uterine rupture in the first and second trimesters and pregnancy outcomes after fertility‐sparing surgery for AOC.

## 2 CASE REPORT

A 31‐year‐old pregnant woman (Gravida 2, Para 0) was admitted to our tertiary perinatal center with acute abdominal pain. The patient was in the 18^th^ gestational week of a dichorionic‐diamniotic twin pregnancy resulting from embryo transfer. The medical history of the patient included a diagnosis of low‐grade endometroid serous‐mucinous AOC.

### 2.1 Oncologic history

Advanced ovarian cancer was primarily detected due to suspicious findings in the right ovary. According to the staging examination, the tumor was staged as initial FIGO (International Federation of Gynecology and Obstetrics) stage IIIC. Due to the patient's strong desire for fertility protection, five oocytes were retrieved and cryopreserved after hormone stimulation. This was followed by primary systemic therapy with three cycles of carboplatin/paclitaxel and bevacizumab for the first and second cycles. After the third cycle, a debulking fertility‐preserving surgery with complete resection of the pelvic and uterine serosa, including cryopreservation of the left ovary, was performed. Postoperatively, the systemic therapy was completed with a further three cycles of carboplatin/paclitaxel and bevacizumab. The subsequent maintenance therapy with bevacizumab was discontinued after the 16th administration due to increasing proteinuria.

### 2.2 Obstetric history

Thirteen months after the last dose of bevacizumab, the patient received an embryo transfer of two embryos resulting in a dichorionic‐diamniotic twin pregnancy. The first‐trimester screening did not reveal any abnormalities. The pregnancy proceeded without any problems until admission to the emergency room. At presentation, the patient described a sudden onset of severe abdominal pain for a few hours, but no vaginal bleeding or discharge. Blood pressure of 105/72 mmHg and heart rate of 88 beats per min were within the normal range, but her temperature was slightly elevated at 37.0°C. Clinical examination revealed a diffuse, defensive tension in the abdomen. On vaginal examination, the cervix was closed.

Sonographic examination discovered an intact intrauterine twin pregnancy and free fluid in the pouch of Douglas. The laboratory tests showed slightly decreased hemoglobin: 11.3 g/dL (normal 12.3–15.3 g/dL) interpreted as pregnancy induced and leukocytes of 13.3 giga/L (normal 4.3–11.3) with neutrophilia of 11.7 × 10^9^ cells/L (normal 1.3–6.7).

**FIGURE 1:**
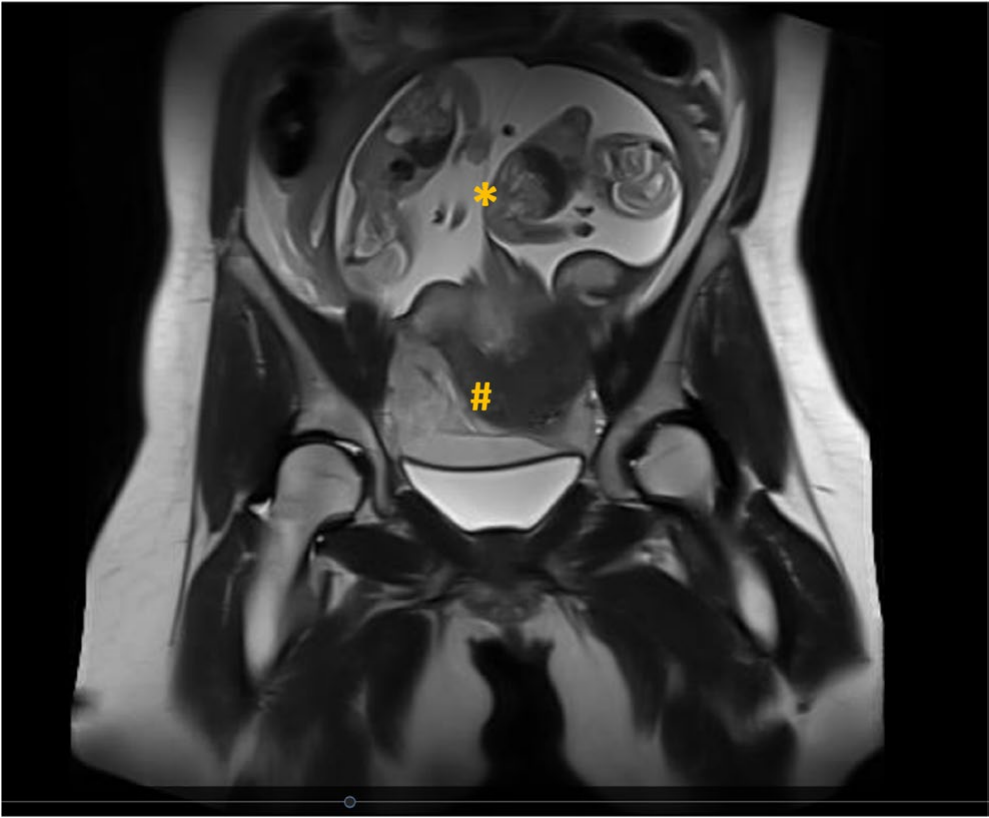
Uterine rupture (#) with the extracavitary position of the twin pregnancy (*).

Acute appendicitis was suspected as the most probable diagnosis and therefore magnetic resonance imaging of the abdomen was indicated. This was performed approximately 1.5 h after admission of the patient and surprisingly revealed a uterine rupture in the fundus area with extra cavitary position of both fetuses (Figure 1).

Simultaneously, the clinical symptoms deteriorated and therefore the decision for emergent abdominal laparotomy was made. Unfortunately, direct preoperative sonography found absent heartbeat in both twins.

**FIGURE 2:**
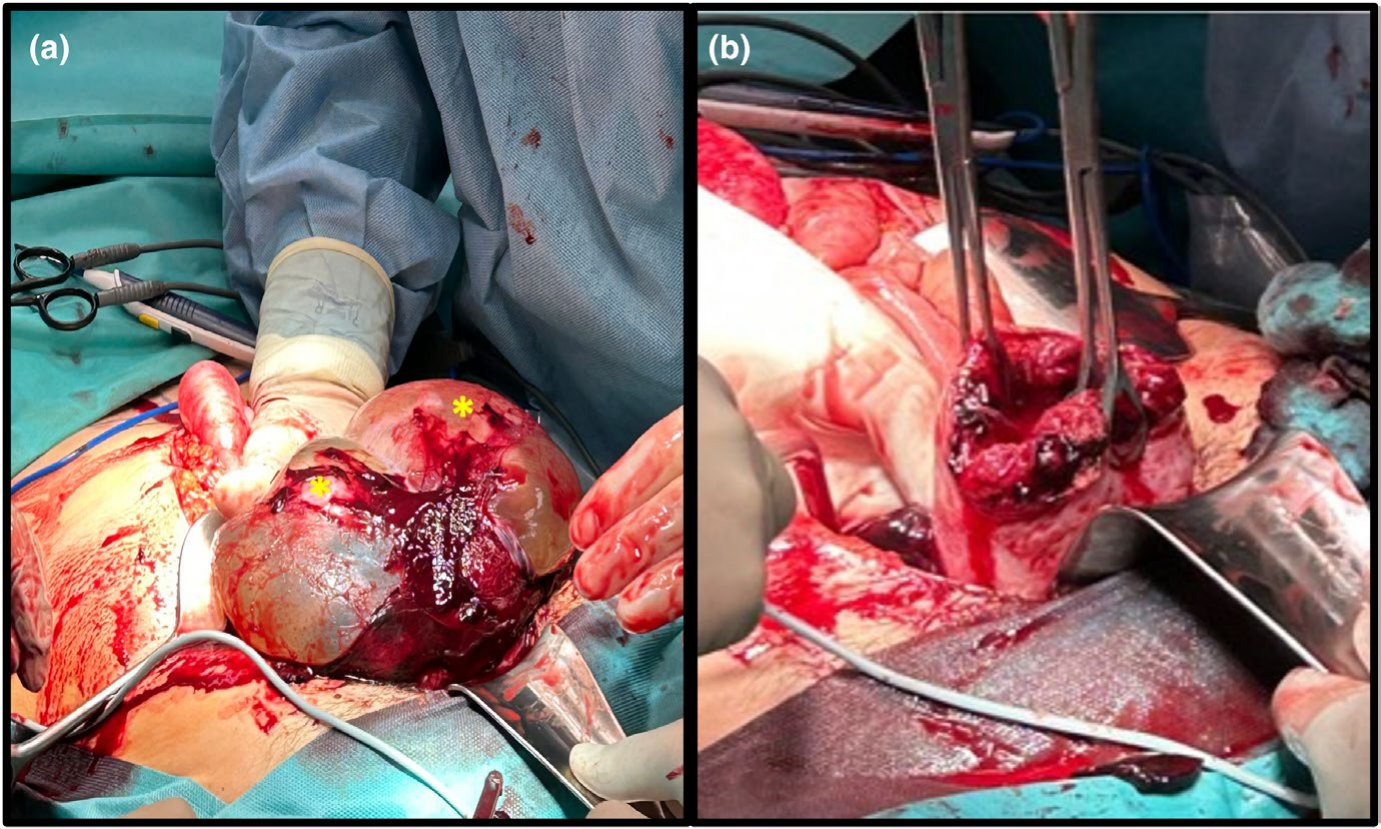
Intraoperative findings. Hematoperitoneum and extrauterine avital fetuses (*) pregnancy (a) with complete uterine rupture in the fundus area (b). Images are taken from the right laterocaudal angle.

After median laparotomy through the abdominal scar, hemoperitoneum was confirmed. Moreover, twins were found in the abdominal cavity (Figure 2a) as the consequence of uterine rupture. After removal of the twins and their placentas and establishing hemostasis we found the uterine rupture atypically in the area of the fundus (Figure 2b). Thereafter, the uterus was reconstructed in two layers. In addition, the abdomen was explored, and biopsies were taken of the anterior uterine wall, serosa of the sigmoid colon and the left and right pelvic wall for histopathologic examination to exclude recurrence of the ovarian cancer. Intraoperatively, a total blood loss was estimated at 4 L and the patient required transfusion of six erythrocyte concentrates, three fresh frozen plasmas, 2 g of fibrinogen and catecholamine support. After surgery, she was transferred to the intensive care unit.

Uterine atony prophylaxis with continuous oxytocin infusion (40 IU in 1000 mL balanced electrolyte solution with 80 mL/h) was continued for 48 h. The patient was extubated the following day without complication and psychosomatic support was provided. Two days after surgery, the patient had improved and was transferred to the gynecology ward, and subsequently discharged 2 days later. Four weeks post‐surgery, the patient presented for the planned follow‐up examination with good postoperative healing.

In order to exclude abnormal invasive placenta, biopsies from the placental bed were taken, but histopathologic examination ruled out this condition. Similarly, the peritoneal biopsies excluded recurrence of ovarian cancer. Finally, as a shared decision, the patient was advised to wait for at least 2 years until the next pregnancy.

## 3 DISCUSSION

Our case report describes two rarities. The first is the possibility of the pregnancy after AOC and the second is an extremely rare spontaneous uterine rupture in the second trimester of pregnancy without any known classic risk factor.

Uterine rupture occurs mainly during the third trimester or labor^5^ and its incidence in unscared uteri is estimated at 0.2 per 10 000 births with the majority occurring during labor.^6^ The most common risk factor for the occurrence of uterine rupture is previous uterine surgery as cesarean section, myomectomy, as well as abnormal invasive placenta or uterine anomalies.^7^, ^8^, ^9^, ^10^ The patient in this report had none of these factors. However, there have been previously published case reports of seven spontaneous uterine ruptures in the first and second trimesters without known risk factors which are summarized in Table 1.^11^, ^12^, ^13^, ^14^, ^15^ Similar to our case, all these patients presented with sudden abdominal pain; in all cases the uterine rupture occurred in the fundal area, and in only one case the unborn could be saved.

**TABLE 1:** Spontaneous uterine rupture in the first and second trimester without known risk factors – literature overview.

Author/year/Year	Country	Study design	Age, gravidity/parity	Pregnancy week	Clinical presentation/localization of the rupture (fundus/isthmus)	Outcome (mother/child)
Retzke et al. 2009^11^	USA	Case report	Not specified, IV Gravida, III Para	17th	Vaginal bleeding, acute abdomen/fundus rupture	Complete repair of the uterine wall defect. Vital mother and fetus
Katwal et al. 2021^12^	Nepal	Case report	23 years, I Gravida, 0 Para	11th	Sudden onset of abdominal pain, multiple episodes of vomiting/fundus rupture	Complete repair of the uterine wall defect. Avital fetus
Park et al. 2005^13^	Korea	Case report	36 years, II Gravida, II Para	10th	Sudden onset of abdominal pain/fundus rupture	Complete repair of the uterine wall defect. Avital fetus
Chen et al. 2023^14^	China	Case report (*n* = 2)	31 years, III Gravida, II Para 28 years, II Gravida, I Para	12th 20th	Sudden onset of abdominal pain/posterior wall rupture/fundus rupture	Complete repair of the uterine wall defect/subtotal hysterectomy. Avital fetuses
Amro & Lotfi, 2019^15^	United Arab Emirates	Case report (*n* = 2)	27 years, IV Gravida, III Para 34 years, IV Gravida, I Para	12th 12th	Sudden onset of abdominal pain and vomiting/fundus ruptures	Complete repair of the uterine wall defect. Avital fetuses

Moreover, there are no published data regarding uterine rupture after resection of the uterine serosa or in pregnant women with a history of ovarian cancer. Due to the rarity of ovarian cancer in women of reproductive age, the literature is scarce on the outcome of pregnancies after fertility‐preserving surgery in these women (Table 2). None of the studies describe uterine rupture,^16^, ^17^, ^18^, ^19^, ^20^ but in general they report on no increased rate of complications during pregnancy and no increased risk in maternal and fetal mortality or morbidity in pregnant women after ovarian cancer.^16^, ^17^, ^18^, ^19^, ^20^

**TABLE 2:** Pregnancy outcomes after fertility‐preserving surgery for ovarian cancer—literature overview.

Author/year/Year/Year	Country	Study design	Patients with fertility preserving surgery (n)	FIGO stage included	Number of patients with adjuvant chemotherapy	Pregnancy outcomes			
						Conception rate	Live birth	Adverse outcome	Others
Bercow et al. 2021^17^	USA	Systematic review	*n* = 4094	Stage IA Stage IB Stage IC	Not specified	50%–93%	65%–96%	Not specified	Recurrence rate: 5%–32%
Ko et al. 2023^18^	Taiwan	Retrospective single‐center study	*n* = 33	Stage IA Stage IC	*n* = 20	85.7%	93.3%	Not specified	Similar pregnancy rates regardless of whether they had prior live birth experience
Nishio et al. 2022^20^	Japan	Retrospective secondary data analysis	*n* = 25	Stage IA Stage IC	*n* = 18	Not specified	100%	No association with increased rates of preterm birth or neonatal morbidity	Higher cesarean delivery rate
Nitecki et al. 2021^19^	USA	Retrospective population‐based analysis	*n* = 154	Stage IA Stage IC	*n* = 40	Not specified	Not specified	No association with increased rates of preterm birth or neonatal morbidity	No increased cesarean delivery rate
Satoh et al. 2010^16^	Japan	Retrospective multi‐institutional analysis	*n* = 211	Stage IA Stage IC	*n* = 125	28.5%	70%	Not specified	Not specified

Abbreviation: FIGO, The International Federation of Gynecology and Obstetrics.

However, only patients with early stage IA–IC of ovarian cancer were included in the analyzed studies. On the other hand, our patient had advanced stage IIIC and there are no studies reporting pregnancy after AOC according to our research.

Regarding systemic treatment, the administered platinum/taxane‐based chemotherapy does not appear to have had an impact on pregnancy outcomes in previous publications.^20^, ^21^, ^22^ On the other hand, bevacizumab, which is an angiogenesis inhibitor, can cause gastrointestinal ruptures due to change in vascular integrity^23^, ^24^ and diaphragmatic and non‐pregnant uterine ruptures during treatment with bevacizumab have been reported.^25^, ^26^, ^27^ Moreover, there are publications describing the effect of bevacizumab on the reduction of smooth muscle,^28^ crucial for its use in the treatment of benign metastatic leiomyomatosis.^29^ Therefore, the bevacizumab could be considered as a relevant factor in our case. Nevertheless, the described ruptures occurred during or shortly after ongoing therapy with bevacizumab.^23^, ^24^, ^25^, ^26^, ^27^, ^30^ In contrast, the uterine rupture in our case did not occur until 17 months after the last bevacizumab administration.

The third possible contributing factor is the twin pregnancy. Only two case reports of uterine rupture in twin pregnancies without the presence of classic risk factors were identified which occurred in the 11th and 30th week of pregnancy.^31^, ^32^ The adenomyosis, which the patients in both cases had, and the overstretching of the uterus due to the twin pregnancy, were considered as crucial factors in these cases. In our case adenomyosis was absent, but rapid extension of the uterus due to twin pregnancy could have been a contributing factor to the rupture.

Ultimately, a clear explanation of our case of uterine rupture in the 18th week of pregnancy is still missing. A combination of the resection of the uterine serosa during the therapeutic operation for ovarian cancer resulting in microlesions of the uterus, which were impaired in healing under therapy with bevacizumab, and the uterine overstretching due to the twin pregnancy could be considered as a multifactorial explanation of the case presented here.

When considering risk of fertility sparing surgery in AOC, the obstetrical complications are relevant, but the oncologic outcome, with the increased risk of recurrence is dominant because it determines the overall survival. Therefore, the current guidelines suggest the possibility of fertility preserving surgery only in early ovarian cancer up to stage IC. There are no recommendations regarding fertility sparing surgery in AOC or optimal timing of pregnancy after AOC.

## 4 CONCLUSION

Pregnancy after ovarian cancer is possible but should be monitored carefully. The probability of uterine rupture without direct surgical intervention in myometrium is an extremely rare condition, but it should be considered as a differential diagnosis in pregnant women with strong acute abdominal pain and fluid in the pouch of Douglas. Further studies could investigate a possible causal relationship between uterine rupture and therapy with bevacizumab.

## AUTHOR CONTRIBUTIONS

Stefan Lukac: Writing, conception, design, interpretation and supervision. Robin Wenzel: Writing, literature review and design. Fabienne Schochter: Supervision, interpretation and critical review. Ulrike Friebe‐Hoffmann: Supervision, interpretation and critical review. Beate Hüner: Supervision, interpretation and critical review. Wolfgang Janni: Supervision, interpretation and critical review.

## FUNDING INFORMATION

No funding.

## CONFLICT OF INTEREST STATEMENT

The authors declare that they have no conflict of interest.

## INFORMED CONSENT

The patient has given her consent for publication of her case. The Ethics committee of Ulm University have also approved the publication of this case report.

## Data Availability

Data sharing is not applicable to this article as no new data were created or analyzed in this study.
